# Supplementary material for: Evaluating the Inclusion of Cold-Pressed Rapeseed Cake in the Concentrate for Dairy Cows upon Ruminal Biohydrogenation Process, Ruminal Microbial Community and Milk Production and Acceptability
Source: Animals (Basel). 2021 Aug 30;11(9):2553. doi: 10.3390/ani11092553 (PMC8471125; doi:10.3390/ani11092553)
Supplement: Supplementary file 1 [file animals-11-02553-s001.zip › Supplementary Table S1.pdf]

Supplementary Table S1. Mean relative abundances of bacterial and Eukaryote phyla in ruminal samples of cows when fed a concentrate formulated with cold-pressed rapeseed cake or a control concentrate

Phylum	Treatment		SEM	P-value
	CTR	CPRC		
**16S rRNA**				
Bacteroidetes[1]	45.7	55.8	5.02	0.1762
Firmicutes[1]	37.6	28.5	4.09	0.1352
Proteobacteria[2]	-0.124	-0.059	0.0519	0.3976
Tenericutes[2]	-0.434	-0.611	0.0547	0.0382
Euryarchaeota[2]	0.105	0.225	0.0772	0.2898
Actinobacteria[2]	-0.532	-0.583	0.0947	0.7076
Chloroflexi[2]	-0.988	-1.031	0.0945	0.7540
Cyanobacteria[2]	-0.973	-0.897	0.0872	0.5465
Planctomycetes[1]	1.31	0.89	0.144	0.0560
Spirochaetes[3]	0.534	0.609	0.1006	0.6090
Sinergistetes[1]	0.152	0.111	0.0174	0.1100
Verrucomicrobia[2]	-0.442	-0.608	0.0719	0.1238
TM7[1]	4.63	3.48	0.685	0.2552
WPS2[2]	-0.587	-0.934	0.1487	0.1214
**18S rRNA**				
Ascomycota[2]	1.42	1.47	0.088	0.7362
Basidiomycota[1]	1.40	0.59	0.479	0.2479
Mucoromycota[1]	2.63	3.08	0.703	0.6625
Neocallimastigomycota[1]	11.5	3.8	2.00	0.0163
Ciliophora[1]	33.7	41.8	7.14	0.4374
Cercozoa[1]	0.25	1.02	0.613	0.3905
Ochrophyta[1]	0.029	0.040	0.0339	0.8302

CTR: Control; CPRC: cold-pressed rapeseed cake; [1]Untransformed data; [2]Log transformation; [3] square root transformation; SEM: standard error of the mean
