# Supplementary material for: Evaluating the Inclusion of Cold-Pressed Rapeseed Cake in the Concentrate for Dairy Cows upon Ruminal Biohydrogenation Process, Ruminal Microbial Community and Milk Production and Acceptability
Source: Animals (Basel). 2021 Aug 30;11(9):2553. doi: 10.3390/ani11092553 (PMC8471125; doi:10.3390/ani11092553)
Supplement: Supplementary file 1 [file animals-11-02553-s001.zip › Supplementary Table S2.pdf]

Supplementary Table S2. Mean relative abundances of bacterial families in ruminal samples of cows when fed a concentrate formulated with cold-pressed rapeseed cake or a control concentrate

Family	Treatment		SEM	P-value
	CTR	CPRC		
Ruminococcaceae[2]	0.90	0.79	0.038	0.0839
Prevotellaceae[1]	37.1	47.6	4.46	0.1194
Paraprevotellaceae[1]	1.01	1.30	0.195	0.3268
Clostridiaceae[1]	0.37	0.27	0.043	0.1299
Lachnospiraceae[2]	1.09	0.93	0.058	0.0808
Veillonaceae[1]	6.07	4.7	0.84	0.2677
RF16[1]	4.6	3.5	0.68	0.2553
Christensenellaceae[2]	-0.45	-0.64	0.057	0.0331
Mogibacteriaceae[2]	-0.25	-0.33	0.058	0.3879
Spirochaetaceae[3]	0.51	0.59	0.103	0.5971
Desulfovibrionaceae[1]	0.14	0.11	0.013	0.1240
Succinivibrionaceae[2]	-0.94	-0.72	0.122	0.2382
Dethiosulfovibrionaceae[1]	0.13	0.10	0.016	0.2183
Erysipelotrichaceae[2]	-0.82	-0.85	0.075	0.7939
Pirellulaceae[1]	1.27	0.84	1.144	0.0555
Acetobacteriaceae[2]	-0.94	-1.04	0.097	0.4664
Anaerolinaceae[2]	-1.04	-1.09	0.104	0.7324
RFP12[2]	-0.72	-0.86	0.077	0.2434
S24-7[1]	1.45	1.33	0.293	0.7592
Coriobacteriaceae[2]	-0.61	-0.70	0.101	0.5384
Methanobacteriaceae[2]	0.05	0.12	0.083	0.1570
Methanomassiliicoccaceae[2]	-1.01	-0.84	0.139	0.3850
BS11[1]	1.53	1.50	0.247	0.9092
WCHB125[2]	-0.90	-1.08	0.084	0.1565
WPS2[2]	-0.587	-0.934	0.1487	0.1214
UF_bacteroidales[1]	4.4	4.0	0.54	0.5688
UF_YS2[1]	0.11	0.12	0.031	0.9226
UF_Clostridiales[2]	0.93	0.71	0.057	0.0160
UF_RF39[2]	-0.47	-0.69	0.061	0.0243

CTR: Control; CPRC: cold-pressed rapeseed cake; [1]Untransformed data; [2]Log transformation; [3] square root transformation; SEM: standard error of the mean
