# Supplementary material for: Evaluating the Inclusion of Cold-Pressed Rapeseed Cake in the Concentrate for Dairy Cows upon Ruminal Biohydrogenation Process, Ruminal Microbial Community and Milk Production and Acceptability
Source: Animals (Basel). 2021 Aug 30;11(9):2553. doi: 10.3390/ani11092553 (PMC8471125; doi:10.3390/ani11092553)
Supplement: Supplementary file 1 [file animals-11-02553-s001.zip › Supplementary Table S3.pdf]

Supplementary Table S3. Mean relative abundances of bacterial genera in ruminal samples of cows when fed a concentrate formulated with cold-pressed rapeseed cake or a control concentrate

Genus	Treatment		SEM	P-value
	CTR	CPRC		
Prevotella[1]	37.1	47.6	4.46	0.1194
Ruminococcus[4]	1.89	1.45	0.142	0.0468
Clostridium[1]	0.31	0.21	0.040	0.0989
Anaerovibrio[1]	0.07	0.09	0.012	0.3404
Succiniclasticum[2]	0.59	0.48	0.073	0.3102
Pyramidobacter[2]	-0.97	-1.16	0.067	0.0717
Treponema[3]	0.51	0.58	0.103	0.6045
Desulfovibrio[1]	0.10	0.08	0.012	0.3026
Acetobacter[2]	-0.95	-1.05	0.096	0.4855
p-75-a5[2]	-1.21	-1.43	0.054	0.0132
Bulleidia[2]	-1.28	-1.41	0.109	0.4216
Mogibacterium[2]	-0.82	-0.91	0.096	0.5429
Shuttleworthia[2]	-0.99	-1.28	0.095	0.0506
Pseudobutyrivibrio[2]	-0.75	-0.91	0.195	0.5877
Lachnospira[2]	-1.45	-1.47	0.195	0.9362
Coprococcus[2]	-0.42	-0.52	0.098	0.4953
Butyrivibrio[1]	5.9	4.8	0.98	0.4197
Blautia[1]	0.10	0.06	0.015	0.0459
SHD231[2]	-1.04	-1.09	0.104	0.7324
YRC22[1]	0.68	0.96	0.158	0.2319
CF231[1]	0.20	0.22	0.042	0.8229
Bifidobacterium[2]	-1.53	-2.06	0.373	0.3310
Vadin CA11[2]	-1.09	-0.81	0.161	0.2315
Methanobrevibacter[2]	0.02	0.09	0.083	0.5507
Methanosphaera[2]	-1.21	-1.07	0.102	0.3411
UG_Coriobacteriaceae[2]	-0.65	-0.76	0.106	0.4999
UG_Bacteroidales[1]	4.4	4.0	0.54	0.5688
UG_BS11[1]	1.53	1.49	0.247	0.9092
UG_RF16[1]	0.04	0.05	0.012	0.7448
UG_S247[1]	1.45	1.33	0.293	0.7592
UG_YS2[1]	0.11	0.12	0.031	0.9226
UG_Paraprevotellaceae[1]	0.13	0.12	0.024	0.6910
UG_Clostridiales[2]	0.94	0.71	0.056	0.0160
UG_ Christensenellaceae[2]	-0.45	-0.64	0.057	0.0331
UG_Clostridiaceae[2]	-1.28	-1.29	0.091	0.9358
UG_Lachnospiraceae[2]	-0.91	-1.16	0.059	0.0110
UG_Ruminococcaceae[4]	0.14	0.19	0.015	0.0344
UG_Veillonaceae[2]	0.035	-0.003	0.0936	0.7783
UG_ Mogibacteriaceae[1]	0.42	0.34	0.042	0.2259
UG_Pirellulaceae[1]	1.26	0.84	0.144	0.0553
UG_Rickettsiales[2]	-1.35	-1.57	0.104	0.1549
UG_ Succinivibrionaceae[2]	-1.02	-0.76	0.127	0.1651
UG_RF16[1]	4.60	3.46	0.683	0.2553
UG_RF39[2]	-0.47	-0.67	0.061	0.0243
UG_ RFP12[2]	-0.72	-0.86	0.077	0.2434
UG_ WCHB125[2]	-0.90	-1.08	0.084	0.1565

CTR: Control; CPRC: cold-pressed rapeseed cake; [1]Untransformed data; [2]Log transformation; [3] square root transformation; [4] reciprocal transformation; SEM: standard error of the mean
