# Supplementary material for: Evaluating the Inclusion of Cold-Pressed Rapeseed Cake in the Concentrate for Dairy Cows upon Ruminal Biohydrogenation Process, Ruminal Microbial Community and Milk Production and Acceptability
Source: Animals (Basel). 2021 Aug 30;11(9):2553. doi: 10.3390/ani11092553 (PMC8471125; doi:10.3390/ani11092553)
Supplement: Supplementary file 1 [file animals-11-02553-s001.zip › Supplementary Table S4.pdf]

Supplementary Table S4. OTUs at genus level significantly different (q<0.05) between rumen samples of cows fed control and concentrate with cold-pressed rapeseed cake

OTU ID	Taxonomy	log2FoldChange*	SE	Adjusted P-value
denovo7988	*Entodinium*	2.74	0.579	<0.001
denovo3176	*Entodinium*	2.10	0.563	0.010
denovo132	*Entodinium*	2.09	0.552	0.009
denovo4456	*Entodinium*	1.97	0.580	0.021
denovo5608	UG_Trichostomatia	1.87	0.580	0.031
denovo312	*Entodinium*	1.66	0.558	0.046
denovo8298	*Ophryoscolex*	1.60	0.528	0.046
denovo8554	UG_Trichostomatia	1.58	0.524	0.045
denovo263	*Entodinium*	1.46	0.500	0.048
denovo1637	UG_Neocallimastigaceae	-1.50	0.518	0.050
denovo8811	*Ophryoscolex*	-1.76	0.579	0.045
denovo5447	UG_Haptoria	-2.13	0.544	0.007

UG: undefined genus; SE: standar error; *significant mean positive log2 fold changes correspond to increased relative abundance in cold-pressed rapeseed cake group and significant mean negative log2 fold changes correspond to decreased relative abundance in cold-pressed rapeseed cake group.
